# Releasing the concept of HLA‐allele specific peptide anchors in viral infections: A non‐canonical naturally presented human cytomegalovirus‐derived HLA‐A*24:02 restricted peptide drives exquisite immunogenicit**y**

**DOI:** 10.1111/tan.13537

**Published:** 2019-04-14

**Authors:** Wiebke C. Pump, Rebecca Schulz, Trevor Huyton, Heike Kunze‐Schumacher, Jörg Martens, Gia‐Gia T. Hò, Rainer Blasczyk, Christina Bade‐Doeding

**Affiliations:** ^1^ Institute for Transfusion Medicine Hannover Medical School Hannover Germany; ^2^ Department of Cellular Logistics Max Planck Institute for Biophysical Chemistry Göttingen Germany

**Keywords:** antigen presentation, HCMV, HLA class I, peptides, T‐cells

## Abstract

T‐cell receptors possess the unique ability to survey and respond to their permanently modified ligands, self HLA‐I molecules bound to non‐self peptides of various origin. This highly specific immune function is impaired following hematopoietic stem cell transplantation (HSCT) for a timespan of several months needed for the maturation of T‐cells. Especially, the progression of HCMV disease in immunocompromised patients induces life‐threatening situations. Therefore, the need for a new immune system that delivers vital and potent CD8+ T‐cells carrying TCRs that recognize even one human cytomegalovirus (HCMV) peptide/HLA molecule and clear the viral infection long term becomes obvious. The transcription and translation of HCMV proteins in the lytic cycle is a precisely regulated cascade of processes, therefore, it is a highly sensitive challenge to adjust the exact time point of HCMV‐peptide recruitment over self‐peptides. We utilized soluble HLA technology in HCMV‐infected fibroblasts and sequenced naturally sHLA‐A*24:02 presented HCMV‐derived peptides. One peptide of 14 AAs length derived from the IE2 antigen induced the strongest T‐cell responses; this peptide can be detected with a low ranking score in general peptide prediction databanks. These results highlight the need for elaborate and HLA‐allele specific peptide selection.

## 1 INTRODUCTION

The formation of the immunological synapse, composed of the HLA molecule, a peptide of pathogenic origin, the T‐cell receptor, the CD8 co‐receptor and among others, CD3 components, is very complex. The activation of a specific cytotoxic T‐cell (CTL) is critically dependent from the recruitment and presentation of a pathogenic peptide, because every single peptide alters the structure of the presented peptide‐HLA (pHLA) complex. Virally infected cells that present viral pHLA complexes can be eliminated and viral replication can be prevented.

Immunocompromised patients are subjected to high risk of infection related mortality in the late recovery phase post‐hematopoietic stem cell transplantation (HSCT). Human cytomegalovirus (HCMV) infection represents one of the main challenges. Although, there is evidence in the last years that CD4^+^ T‐cells, NK cells, and B‐cells play a role in HCMV‐specific immunity with different impact, CD8^+^ T‐cells are the key players to protect against HCMV.^1^ The transfer of HCMV specific CD8^+^ T‐cells has shown promising results in combating HCMV, as well as in establishing a protective immunity. The importance of these cells becomes apparent when the T‐cell response is impaired, for example, after HSCT or in patients with genetic or acquired immunodeficiency syndrome.^2^, ^3^, ^4^, ^5^

Although, HCMV has evolved many immune evasion strategies intermittent sub‐clinical reactivations can be controlled by the immune system of healthy individuals. However, HCMV infections continue to be a major problem following allogeneic HSCT,^6^ because the virus has a high prevalence in the population and establishes latency.^7^, ^8^ Despite the development of new techniques in recent years HCMV infection/reactivation and progression to HCMV disease in immunocompromised patients can still become a life‐threatening situation.^9^

One of the main problems for high‐risk patients is the lack of different antiviral drugs directed against different viral proteins. Only few different licensed drugs are currently available for the therapy of HCMV infections and diseases, targeting three different viral proteins,^10^, ^11^, ^12^, ^13^ yet, development of resistance is increasing.^14^, ^15^, ^16^, ^17^ In addition, these drugs have heavy side effects, including myelosuppression which make their administration post HSCT extremely complicated.^18^, ^19^, ^20^, ^21^ Even if an HCMV infection can be prevented in the first month post‐HSCT, late HCMV infection might still occur after discontinuing the antiviral treatment.^22^, ^23^, ^24^

One reason HCMV reactivation is the depletion of T‐cells in the graft to prevent Graft‐vs‐Host‐Disease.^25^, ^26^, ^27^, ^28^, ^29^ Thus, for a timespan of at least several months that is required for the maturation of precursor cells the recipient has an extremely high risk to contract an HCMV infection.^30^ Even in the presence of mature T‐cells, maturated in the recipient or present in the transplant prior to transplantation, HCMV infections still represent an unpredictable risk factor, because it seems like HCMV‐specific T‐cells only expand in the presence of antigen presumably derived from sporadically reactivating viruses.^31^

A highly promising approach is the adoptive transfer of HCMV‐specific T‐cells to infected patients as first reported by Riddell et al^32^ This technique was improved in the last years leading to T‐cell selection based on Interferon gamma (IFN‐γ) secretion or peptide‐HLA multimer binding following antigen‐stimulation.^33^, ^34^, ^35^ For these techniques, it is imperative to know which viral peptides are recruited and subsequently presented on HCMV infected cells. Most peptides that have been studied are derived from the well‐characterized phosphoprotein (pp)65 or the immediately‐early (IE)1 protein, but not in all individuals responses against these two proteins are immunodominant.^36^, ^37^ Best studied are the pp65 peptides NLVPMVATV and TPRVTGGGAM restricted to HLA‐A*02:01 and HLA‐B*07:02, respectively, that usually induce strong T‐cell responses.^38^, ^39^, ^40^, ^41^, ^42^, ^43^ However, the majority of these and other applied peptides are predicted by computational analysis.^36^, ^44^, ^45^ It remains questionable if these peptides would ever be recruited through the patients peptide loading complex, selected by one HLA subtype and naturally presented by HCMV infected cells. This might be an explanation for some unsuccessful T‐cell transfers.^34^, ^46^, ^47^

The challenges with selecting peptides from prediction tools are (a) peptide prediction tools are based on peptide data that have been generated from healthy cells, (b) peptide prediction tools are based on the assumption that the length should be restricted to 9 AA in length, (c) the alteration of the proteome under pathological conditions is not considered. Several studies described peptides of unconventional binding mode, properties, and/or length under pathological conditions.^48^, ^49^, ^50^, ^51^

To establish a reliable system for peptide selection, we utilized soluble (s)HLA‐technology for viral‐peptide fishing in HCMV infected cells.^52^ Our study provides new insights into the origin and biochemical nature of viral peptides that are naturally recruited in HCMV infected cells. Our investigations concentrated on the common allele HLA‐A*24:02. We previously showed the HLA‐A*24:02 restricted peptide repertoire and the biophysical interaction with the peptide loading complex.^53^ Tapasin, an essential protein of the peptide loading complex that facilitates the binding of high affinity peptides, is one of the target structures of HCMV immunoevasions, therefore, the tapasin‐independent peptide recruitment strategy of HLA‐A*24:02 makes this common allele a perfect molecule for the analysis of naturally presented viral peptides. The comprehensive analysis of a common HLA allele that constitutively present viral peptides during all stages of infection will help to improve T‐cell therapies long term even for patients with virus susceptible HLA haplotypes.

## 2 MATERIALS AND METHODS

### 2.1 Cells and viruses

Parental TAP deficient *T2* cells (ATCC, Manassas, Virginia) as well as recombinant HLA‐A*24:02 transduced *T2* cells were cultured in RPMI 1640 (Life Technologies, Darmstadt, Germany) supplemented with 10% fetal bovine serum (FBS, heat inactivated) and 2 mM L‐glutamine (c‐c Pro, Oberdorla, Germany). *HEK293T‐*cells were cultured for transfection in DMEM (Life Technologies, Darmstadt, Germany) supplemented with 10% FBS (heat inactivated), 2 mM L‐glutamine and 1 mg/mL Geneticin (Life Technologies, Darmstadt, Germany). *NHDF‐c* cells were cultured in DMEM supplemented with 10% FBS (heat inactivated) and 2 mM L‐glutamine. Parental fibroblast *BJ* cells (ATCC, Manassas, Virginia) as well as recombinant HLA‐A*24:02 transduced *BJ* cells were cultured in DMEM supplemented with 20% medium 199, 10% FBS (heat inactivated) and 2 mM L‐glutamine. All HCMV infections were performed with strain AD169 (ATCC). For the preparation of virus stock, *NHDF‐c* ells (PromoCell, Heidelberg, Germany) were infected and cultured until 100% cytopathic effect was seen. The virus was pelleted and stored at −80°C. To determine the virus titer, plaque‐assay was performed on *NHDF‐c* cells cultured in carboxymethyl cellulose (CMC) medium.

### 2.2 Peptides

All peptides were purchased from Thermo Fisher (Waltham, Massachusetts) and dissolved in DMSO.

### 2.3 Lentiviral transduction of human fibroblasts and lymphocytes

The lentiviral vectors encoding for membrane‐bound (m) or soluble (s) HLA‐A*24:02 were generated as previously described.^53^ Transduction of *T2* or *BJ* cells with pRRL.PPT.SFFV.mcs.pre/mHLA‐A*24:02 or pRRL.PPT.SFFV.mcs.pre/sHLA‐A*24:02, respectively, was performed as described.^54^ Expression of sHLA‐A*24:02 was varified by sandwich‐ELISA using mAb W6/32 (AbD Serotec, Puchheim, Germany) for coating and anti‐β2m‐HRP as detection antibody. Transduction was verified by assessing mRNA levels of HLA‐A*24:02 with quantitative real‐time PCR (qRT‐PCR) and protein level via flow cytometry with an anti‐A9‐FITC mAb (OneLambda, Kittridge, California).

### 2.4 Large‐scale production of sHLA‐A*24:02 molecules in HCMV infected fibroblasts

For large‐scale production of sHLA molecules BJ/sHLA‐A*24:02 cells were cultured in a bioreactor. After 1 week, the cells were infected with AD169 at a multiplicity of infection (MOI) of 1. The supernatant containing sHLA molecules was harvested weekly. Because HCMV infected cells die off, healthy uninfected BJ/sHLA‐A*24:02 cells were introduced into the bioreactor along with fresh medium. Protein production and infectiosity of the supernatant was validated weekly. To monitor protein production, sandwich‐ELISA was performed. To test infectiosity, healthy *BJ* cells were infected with the supernatant and checked for cytopathic effects. Affinity purification of the sHLA molecules was performed as detailed elsewhere.^55^ In brief, supernatant from several weeks was pooled and purified on an NHS‐(N‐hydroxysuccinimide)‐activated *HiTrap* column coupled to mAb W6/32. The elution of sHLA‐peptide complexes was performed with 0.1 M Glycine/HCL buffer at pH 2.7.

### 2.5 Mass spectrometric analysis of HLA‐A*24:02 bound peptides

We used 3 mg pure HLA‐A*24:02 protein with and without viral treatment. As described by Badrinath et al,^56^ low affinity peptides were separated from HLA heavy chain and beta‐2‐microglobulin (β2m) by size‐exclusion filtration using a 10‐kD cutoff membrane (Millipore, Schwalbach, Germany). The retentate was treated with 0.1% trifluoroacetic acid (TFA) (J. T. Baker, Phillipsburg, New Jersey) followed by size‐exclusion filtration to elute high affinity peptides. The LC/MS analysis was performed with a Dionex UltiMate 3000 high‐performance LC system and an LTQ Orbitrap Velos Hybrid FT Mass Spectrometer. Peptide identification was performed by UniProt database search.

### 2.6 HLA‐A*24:02 binding assay

To find the saturating concentration of the peptides, 5*10 ^*5*^ T2/mHLA‐A*24:02 cells were incubated with different amounts of peptide and β2m (Sigma‐Aldrich, Munich, Germany) in AIM‐V serum‐free medium (Life Technologies, Darmstadt, Germany) at 37°C for 16 hours. Peptide‐loaded T2/mHLA‐A*24:02 cells were stained with anti‐A9‐FITC (One Lambda, Kittridge, California) and analyzed by flow cytometry with a FACS Canto II (BD, Heidelberg, Germany). T2/mHLA‐A*24:02 cells incubated with β2m, but without peptide served as negative control and cells loaded with the HCMV pp65 peptide VYALPLKML (VL9), reported to stimulate CD8^+^ T‐cells,^45^ served as positive control.

### 2.7 Collection of PBMCs

Written informed consent was obtained from all donors, as approved by the Ethics Committee of Hannover Medical School. Blood was taken from healthy donors, typed for HLA‐A*24:02 and tested CMV seropositive (Table 1). PBMCs were isolated by density gradient centrifugation with Lymphosep (c.c.pro GmbH, Oberdorla, Germany).

**Table 1:** HLA class I genotypes and CMV status of healthy donors that were used for PBMC isolation

PBMCs from individual	HLA class I genotype	CMV status
**A**	A*24:02 A*32:01 B*08:01 B*40:01 C*01:02 C*07:01	Seropositive
**B**	A*11:01A*24:02B*13:02B*55:01C*03:03C*06:02	Seropositive
**C**	A*24:02A*26:01B*15:01B*39:01C*03:03C*12:03	Seropositive

### 2.8 T‐cell stimulation

The PBMCs were incubated for 1 hour at 37°C in RPMI/10% AB serum. Afterwards, one half of the cells (stimulators) was mixed with 10 μg of peptide, incubated for 1 hour at 37°C and irradiated at 30 Gy. Those cells were incubated with the other half of the PBMCs (responders) in RPMI/10% AB serum with IL‐2 at 37°C for 14 days. On day 3 and 10, IL‐7 was added and on day 5 and 12 IL‐2 was added. On day 7 the cells were restimulated with fresh peptide loaded, irradiated PBMCs.

### 2.9 T‐cell characterization and cytotoxic potential

On day 0, 7, and 14, the PBMCs from the T‐cell stimulation assay were analyzed in flow‐cytometry by anti‐CD3‐FITC, CD8‐PerCP, CD45RO‐PE, CD45RA‐APC‐H7, and CD69‐APC antibodies (BD, Heidelberg, Germany) with a FACS Canto II.

Cytotoxicity of effector cells that were stimulated for 14 days was tested by measuring LDH release with the CytoTox 96 Non‐Radioactive Cytotoxicity Assay (Promega, Madison, Wisconsin) following incubation with peptide loaded T2/mHLA‐A*24:02 cells at a ratio of 5:1. The cells were incubated for 4 hours at 37°C and LDH release was measured according to kit instructions.

### 2.10 Inversed peptide search

For completeness, we surveyed peptide databases for the naturally presented HCMV‐derived peptide AQ14; two databases that include HLA‐allele specific 14‐meric peptides were secreened: IEDB database and NetMHCpan.^57^ The MHC I binding predictions were made on 2/5/2018 using the IEDB analysis resource Consensus tool^58^ that combines predictions from ANN,^59^, ^60^ SMM^61^ and comblib.^62^

### 2.11 Structure modeling

Rosetta Flexpepdock was used for peptide docking using the structures of HLA‐A*24 3WL9 and a 15‐meric peptide derived from the HLA‐A*02:01 4U6Y crystal structure. A total of 200 models were created and then ranked based on their Rosetta generic full‐atom energy score.

## 3 RESULTS

### 3.1 HCMV infected cells show an altered peptide repertoire

The first objective of this study was to analyze which peptides would be selected and presented by HLA‐A*24:02 in HCMV infected fibroblasts during the replication phase of HCMV. Therefore, BJ cells were transduced with the extracellular domain of HLA‐A*24:02 and infected with HCMV. To monitor sHLA‐A*24:02 expression the cell supernatant was analyzed by ELISA over time. A distinct alteration in the presence of sHLA‐A*24:02 expression levels could be observed following infection (Figure S1 ). To ensure BJ cells were infected, the cells were tested for pp65 expression through flow cytometry and the supernatant was tested for infectivity.

The pp65 expression of HCMV‐infected cells varies during infection because it is transcribed from a late phase gene. On day 7 post‐infection, the cells showed a cytopathogenic effect (CPE) (Figure S2 ), the bioreactors were harvested, an estimated 70% of the cells were dead and approximately 50% expressed pp65 (Figure S3). From this, we inferred that these cells were infected by HCMV and already released viral particles to infect the remaining cells.

**Figure 1:**
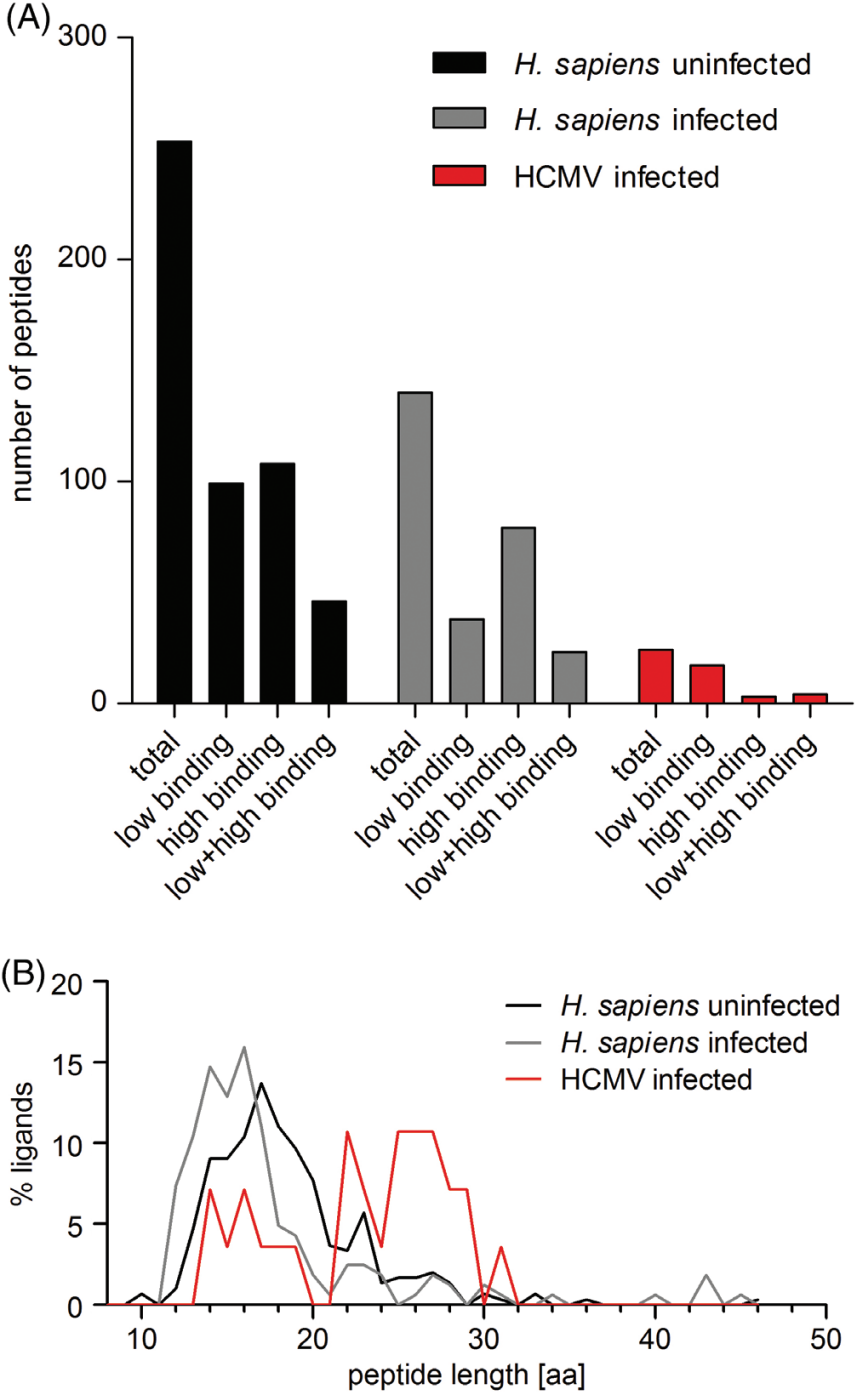
Characteristics of identified peptides from HLA‐A*24:02. Recombinant fibroblasts expressing soluble HLA‐A*24:02 molecules infected with human cytomegalovirus (HCMV) strain AD169. The control cells were uninfected. The soluble peptide/HLA‐A*24:02 complexes were purified by affinity chromatography. Low binding peptides were separated from HLA by size‐exclusion filtration; high binding peptides were eluted from HLA by treatment with 0.1% trifluoroacetic acid (TFA) and subsequently separated by size‐exclusion filtration. Peptides derived from HCMV and Homo sapiens were studied separately. Each peptide sequence was counted once for each category, regardless whether it was detected several times or not. A, Number of isolated peptides from different conditions. B, Length distribution of HLA‐A*24:02 bound peptides. aa: amino acid; H. sapiens uninfected: H. sapiens‐derived peptides, uninfected cells; H. sapiens infected: H. sapiens‐derived peptides, HCMV infected cells; HCMV infected: HCMV‐derived peptides, HCMV infected cells

The cell culture supernatant containing the soluble peptide‐HLA complexes was collected and peptide‐HLA complexes were purified by affinity chromatography. Low binding peptides were separated from the HLA molecules directly by size‐exclusion filtration. High binding peptides were eluted from the HLA molecules by treatment with 0.1% TFA and subsequently separated by size‐exclusion filtration. In total, we discovered 164 different peptides for HCMV infected and 253 for uninfected BJ/sHLA‐A*24:02 cells (Figure 1A). From the uninfected cells, 99 exclusively low binding and 108 exclusively high binding peptides were found and 46 peptides that were present in both fractions could be recovered. For the infected cells, 140 were derived from *H. sapiens* proteins and 24 different peptides totally matched the protein sequence of HCMV AD169; 17 HCMV‐derived low binding, 3 HCMV‐derived high binding peptides and 4 peptides that were found in both fractions. Of 140 *H. sapiens* derived peptides in HCMV infected cells, 38 peptides were low‐binder, 79 were high‐binder, and 23 were present in both fractions.

The length of the peptides bound to sHLA‐A*24:02 in HCMV infected and uninfected cells was variable and some differences could be observed between peptides from different conditions (Figure 1B). The amino acid length of peptides from human proteins was skewed towards shorter peptides in infected cells compared to uninfected cells, while HCMV‐derived peptides were spread over a broad range of different length. It appears like the shift of human peptides to shorter length in infected cells occurs from the dominant presentation of longer HCMV‐derived peptides.

**Figure 2:**
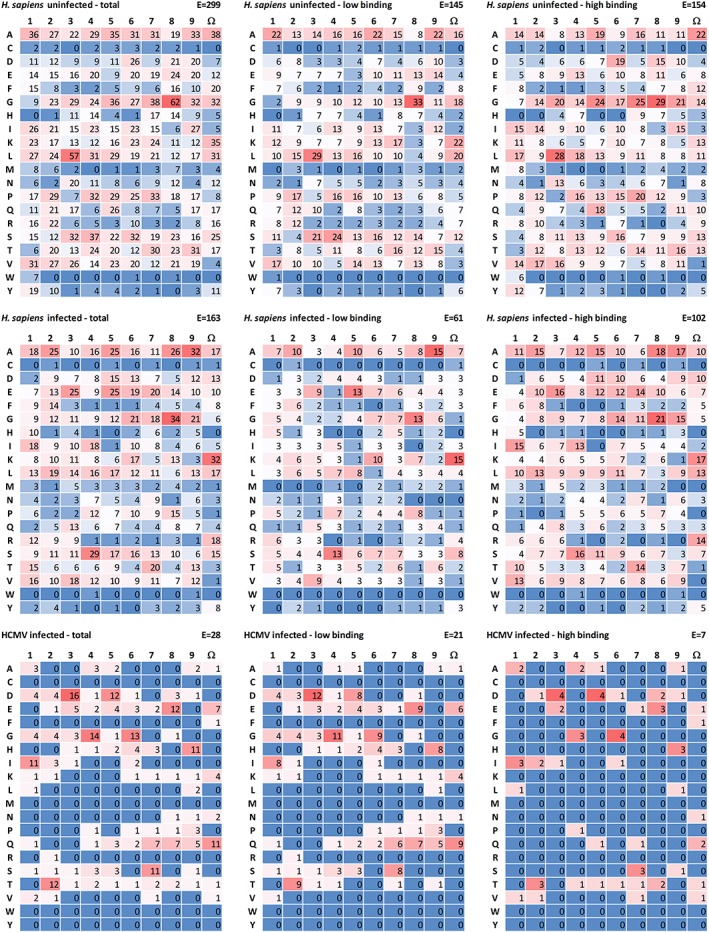
Peptide binding motif of peptides bound to HLA‐A*24:02. For each condition the number of each amino acid at peptide positions 1‐9 and pΩ was determined. Peptides derived from human cytomegalovirus (HCMV) and Homo sapiens were studied separately. Color coded is the number of peptides with that amino acid at that position with the highest in red and the lowest in blue. H. sapiens uninfected: H. sapiens‐derived peptides, uninfected cells; H. sapiens infected: H. sapiens‐derived peptides, HCMV infected cells; HCMV infected: HCMV‐derived peptides, HCMV infected cells

The majority of peptides, regardless if they were isolated from uninfected or HCMV infected cells, had no distinct anchor positions (Figure 2), unlike the published data for HLA‐A*24:02 in B‐lymphocytes.^53^, ^63^, ^64^

**Figure 3:**
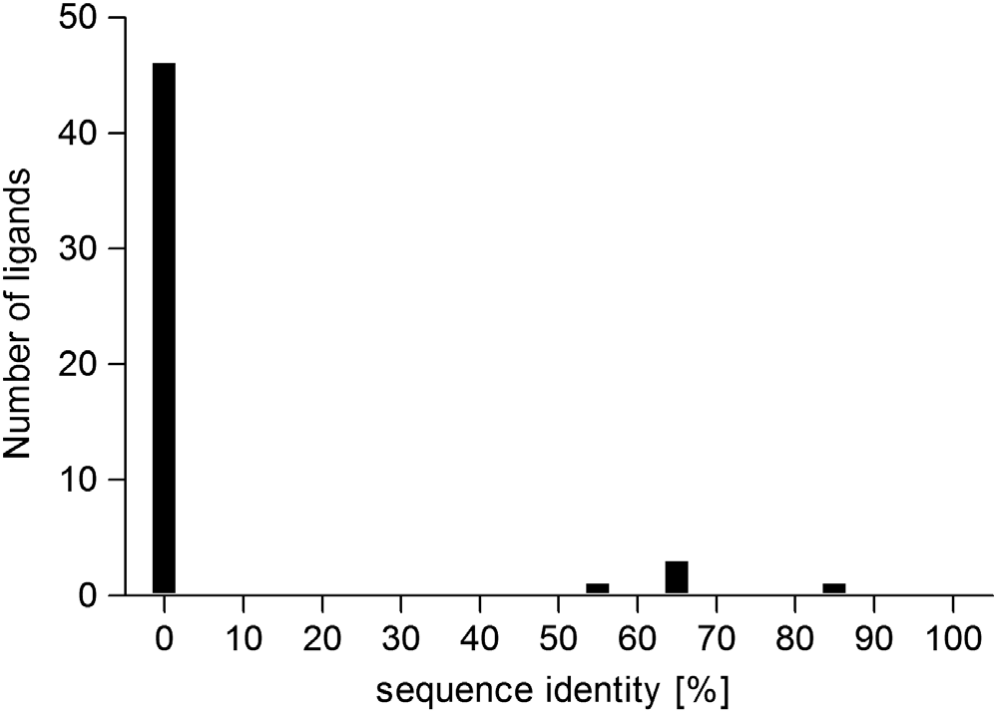
Sequence identity of human cytomegalovirus (HCMV) peptides and *Homo.sapiens* proteins. The identified HCMV peptides were compared to the H. sapiens proteome in the NCBI database, also regarding I/L ambiguity match

All HCMV‐derived peptides were analyzed for sequence identity with *H. sapiens* proteins in the NCBI database, either as exact match or with isobaric Ile/Leu ambiguity match (Figure 3). The exchange of Ile with Leu and vice versa led to a total number of 51 peptides. Only 5 peptides showed little sequence identity with the *H. sapiens* sequence, 1 with 88% and 4 with 60% to 69% sequence identity. All other peptides showed no sequence identity with *H. sapiens* at 100% query coverage.

Strikingly, 19 HCMV peptides were derived from a 31 aa long region at the C‐terminal end of Glycoprotein UL22A (Tables 2 and 3). Only 5 peptides were derived from other proteins: IE2, IRL10, pp85, UL37 homolog, and pp150. The peptides of the IRL10 and IE2 proteins are also derived from the C‐terminal end, whereas the peptides of pp85 and the UL37 homolog are rather from the N‐terminal parts of the proteins and the peptide of pp150 was derived from the middle of the protein.

**Table 2:** HLA‐A*24:02 restricted low binding human cytomegalovirus (HCMV) peptides from AD169 infected BJ cells

Sequence	Abbreviation	Length	Source
AIEAAIQDLRNKSQ	AQ14	14	Viral transcription factor IE2 (strain AD169)
VRETGGTGAAKKPSEK	VK16	16	Phosphoprotein 85 (strain AD169)
KTDEHKENQAKENEKKIQ	KQ18	18	Glycoprotein UL22A (strain AD169)
QKTDEHKENQAKENEKKIQ	QQ19	19	Glycoprotein UL22A (strain AD169)
*ITDGDGSEHQQPQKTDEHKENQ*	IQ22	22	Glycoprotein UL22A (strain AD169)
GDGSEHQQPQKTDEHKENQAKE	GE22	22	Glycoprotein UL22A (strain AD169)
ITDGDGSEHQQPQKTDEHKENQA	IA23	23	Glycoprotein UL22A (strain AD169)
DGSEHQQPQKTDEHKENQAKENE	DE23	23	Glycoprotein UL22A (strain AD169)
GDGSEHQQPQKTDEHKENQAKENE	GE24	24	Glycoprotein UL22A (strain AD169)
SEHQQPQKTDEHKENQAKENEKKIQ	SQ25	25	Glycoprotein UL22A (strain AD169)
ITDGDGSEHQQPQKTDEHKENQAKE	IE25	25	Glycoprotein UL22A (strain AD169)
DGDGSEHQQPQKTDEHKENQAKENE	DE25	25	Glycoprotein UL22A (strain AD169)
GSEHQQPQKTDEHKENQAKENEKKIQ	GQ26	26	Glycoprotein UL22A (strain AD169)
*ITDGDGSEHQQPQKTDEHKENQAKEN*	IN26	26	Glycoprotein UL22A (strain AD169)
*ITDGDGSEHQQPQKTDEHKENQAKENE*	IE27	27	Glycoprotein UL22A (strain AD169)
DGDGSEHQQPQKTDEHKENQAKENEKK	DK27	27	Glycoprotein UL22A (strain AD169)
GDGSEHQQPQKTDEHKENQAKENEKKIQ	GQ28	28	Glycoprotein UL22A (strain AD169)
ITDGDGSEHQQPQKTDEHKENQAKENEK	IK28	28	Glycoprotein UL22A (strain AD169)
ITDGDGSEHQQPQKTDEHKENQAKENEKK	IK29	29	Glycoprotein UL22A (strain AD169)
DGDGSEHQQPQKTDEHKENQAKENEKKIQ	DQ29	29	Glycoprotein UL22A (strain AD169)
ITDGDGSEHQQPQKTDEHKENQAKENEKKIQ	IQ31	31	Glycoprotein UL22A (strain AD169)

Peptides present in low and high binding fraction in italic.

**Table 3:** HLA‐A*24:02 restricted high binding HCMV peptides from AD169 infected BJ cells

Sequence	Abbreviation	Length	Source
*AIEAAIQDLRNKSQ*	AQ14	14	Viral transcription factor IE2 (strain AD169)
AVDAQDVTASAVRAF	AF15	15	Capsid assembly protein UL37 homolog (strain AD169)
VDITDTETSAKPPVTT	VT16	16	Large structural phosphoprotein (strain AD169)
LIEPTGTDDEEDEDDNV	LV17	17	Protein IRL10 (strain AD169)
*ITDGDGSEHQQPQKTDEHKENQ*	IQ22	22	Glycoprotein UL22A (strain AD169)
*ITDGDGSEHQQPQKTDEHKENQAKEN*	IN26	26	Glycoprotein UL22A (strain AD169)
*ITDGDGSEHQQPQKTDEHKENQAKENE*	IE27	27	Glycoprotein UL22A (strain AD169)

Peptides present in low and high binding fraction in italic.

UL22A encodes a CC chemokine RANTES decoy receptor that functions as an immune modulator and is transcribed in the early‐late phase of the viral replication cycle. IE2 is a well‐known transcription factor encoded by UL122 and transcribed in the IE phase to regulate viral early and late gene transcription. IRL10 is a structural envelope glycoprotein of the virus particle. The time of transcription of the IRL10 gene is not known. The tegument proteins pp65, pp150, and UL37 homolog are transcribed from their genes UL25, UL32 and UL47, respectively, in the late phase. The majority of viral peptides that were found on HLA‐A*24:02 are derived from early‐late phase gene products, since most peptides were derived from UL22A. Nevertheless, peptides from other phases of the replication cycle were also presented.

The origin of human‐derived peptides in HCMV‐infected and uninfected cells is partially overlapping and overlap proteins showed a tendency towards a higher number of peptides being presented.

### 3.2 Functional assays revealed differences between the HCMV‐derived peptides

All HCMV‐derived peptides were synthesized and tested for binding to HLA‐A*24:02 in peptide loading assays.

**Figure 4:**
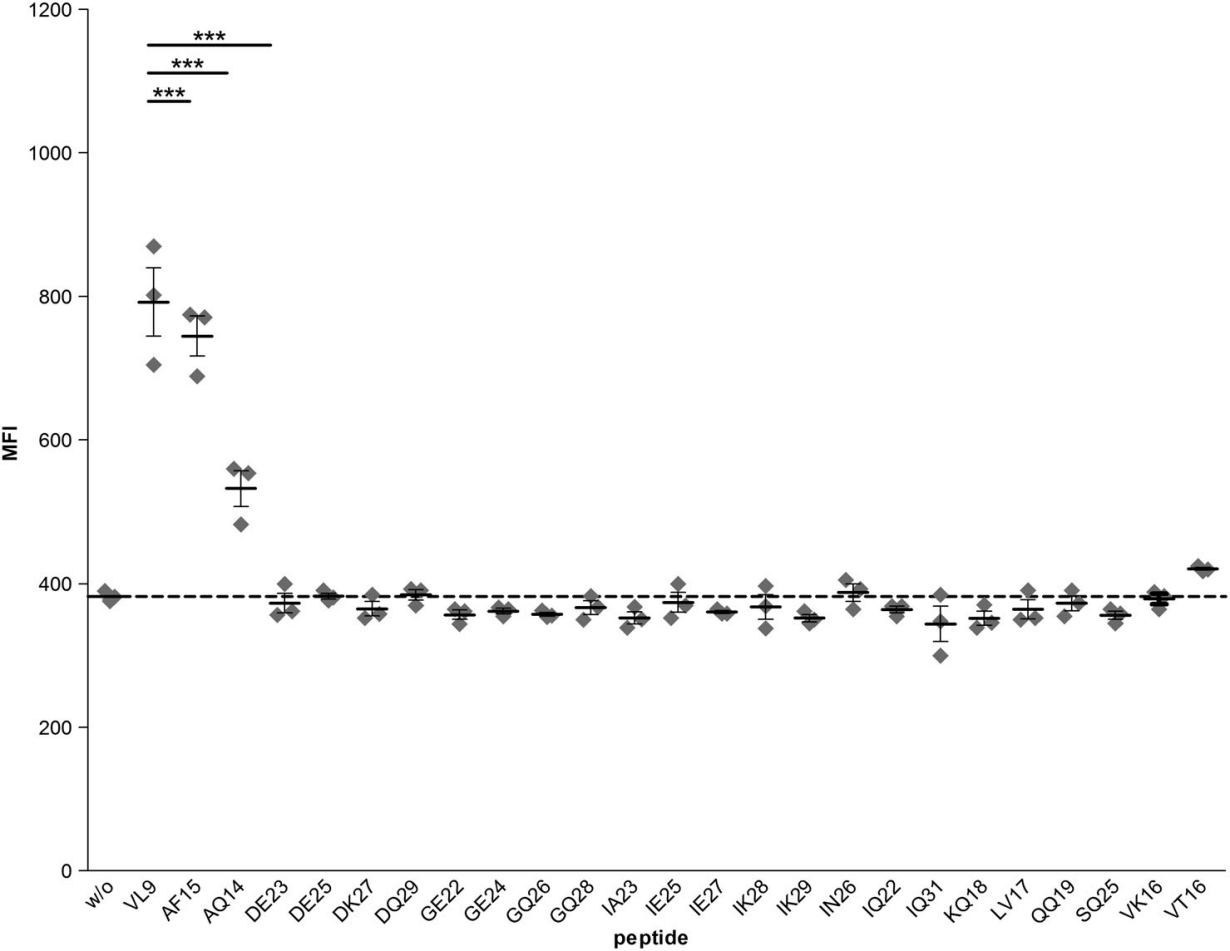
Binding of identified human cytomegalovirus (HCMV)‐derived peptides to *HLA*‐A*24:02 presented by B‐cells. Peptides were incubated with recombinant T2 cells expressing membrane bound HLA‐A*24:02. Peptide binding was measured by utilizing anti‐A9‐FITC antibodies in flow cytometry. Results are representative of three independent experiments with two samples each. Mean fluorescence was compared to transduced T2 cells without peptide. Mean ± SD

pRRL.PPT.SFFV.mcs.pre/mHLA‐A*24:02 transduced T2 cells were separately incubated with all HCMV‐derived peptides and binding was confirmed by flow cytometry (Figure 4). Only 2 of 24 peptides showed significant and efficient binding to T2/mHLA‐A*24:02 cells: AF15 and AQ14. Therefore, the following assays were performed only with these two peptides and the previously described peptide VL9^45^ as positive control.

We loaded PBMCs of HLA‐A*24:02 positive, CMV seropositive individuals with one of the peptides and incubated them with untreated autologous PBMCs for 14 days.

**Figure 5:**
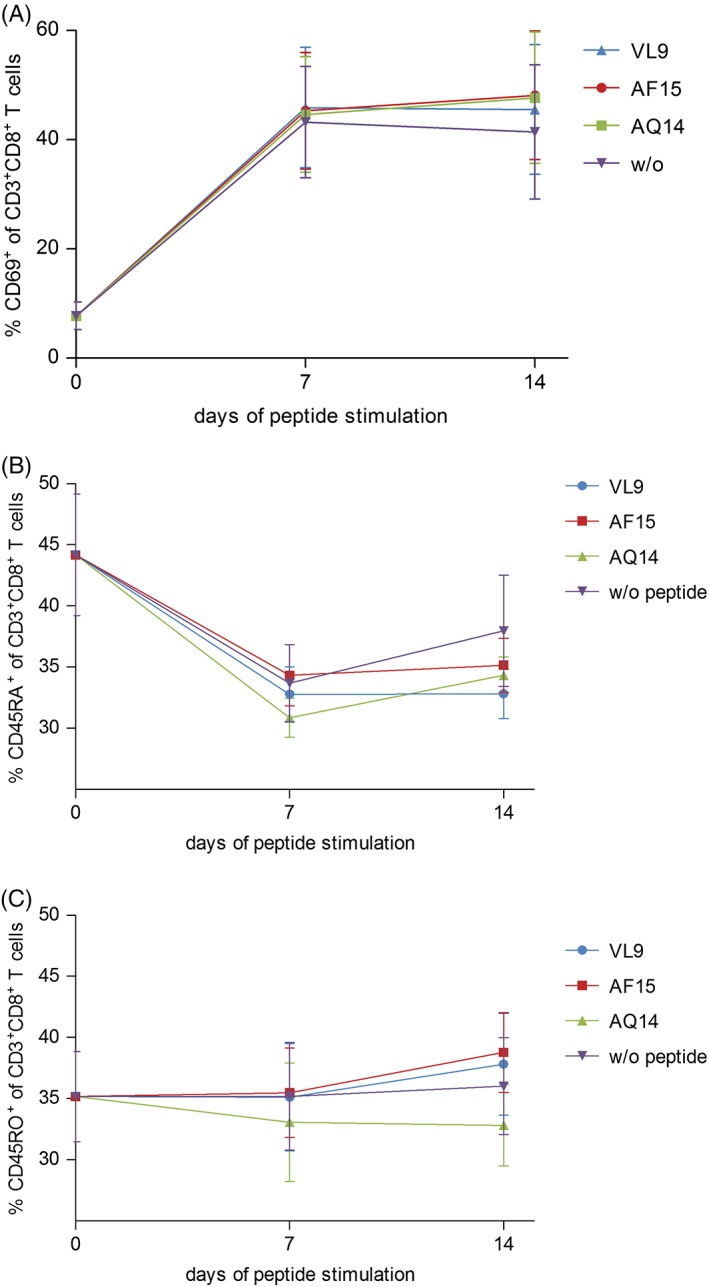
Comparison of markers expressed by CD3^+^CD8^+^ T‐cells following stimulation with different peptides. PBMCs were stimulated with one of three different peptides or only with the solvent for 14 days and expression of CD69 (A), CD45RA (B) and CD45RO (C) CD3^+^CD8^+^ T‐cells was analyzed on day 0, 7, and 14. Results are duplicates from three different individuals. Mean ± SD

On day 0, 7, and 14, the expression of certain CD8^+^ T‐cell markers of was tracked by flow cytometry. Surprisingly, no differences in the expression of the early activation marker CD69, the naïve T‐cell marker CD45RA and the memory T‐cell marker CD45RO could be observed under different stimulation conditions (Figure 5A and B). CD69 was upregulated in all cells post‐stimulation, CD45RA expression decreased and CD45RO expression slightly increased or remained on the same level over 14 days after antigen experience.

**Figure 6:**
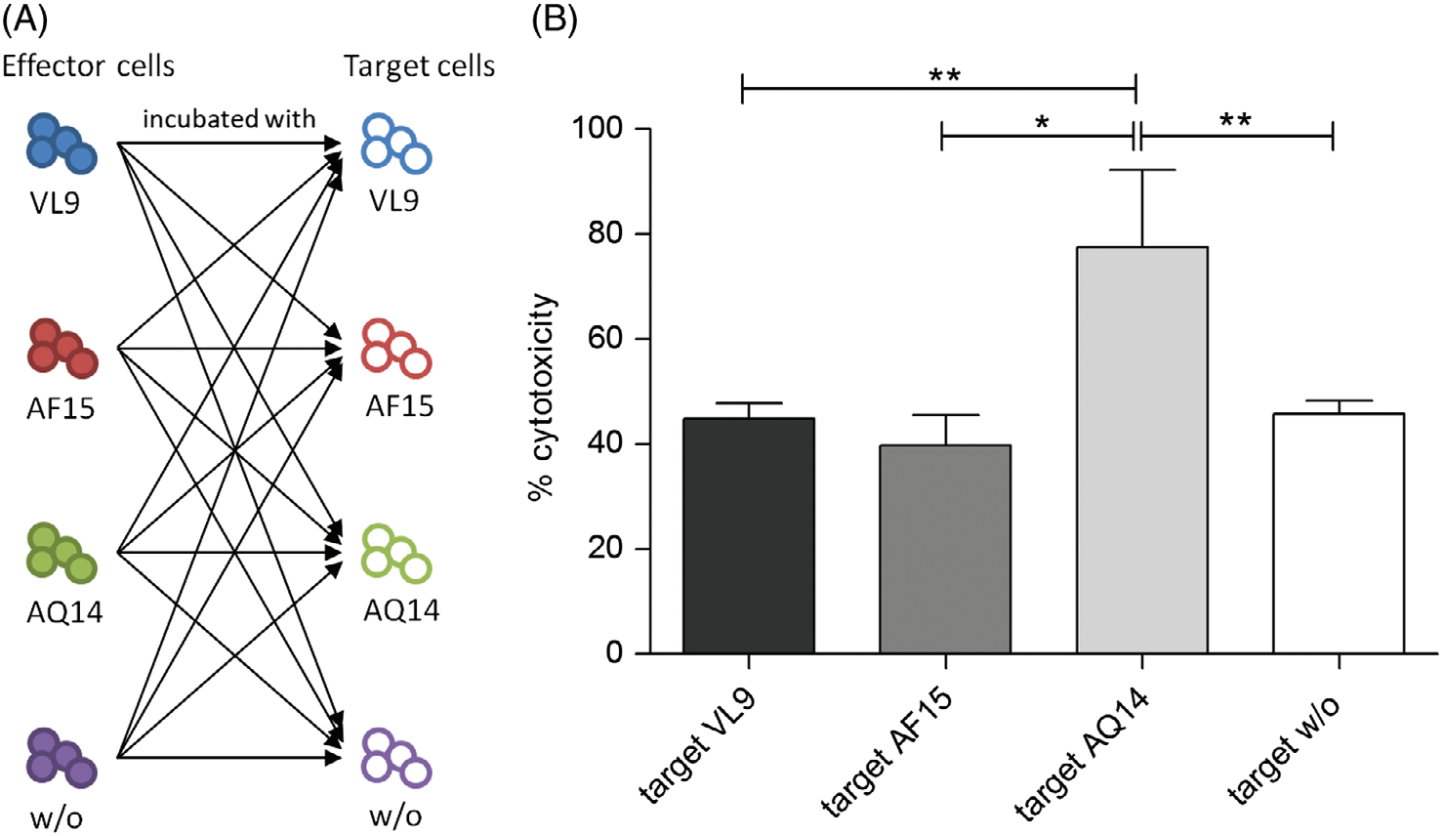
Cytotoxicity of effector cells against peptide loaded B cells after 14 days stimulation. (A) Schematic accomplishment of the experiment. Effector cells of three individuals were stimulated with one of three different peptides or only with the solvent for 14 days to obtain 12 different effector cell cultures. In the next step, each effector cell culture was challenged with all different targets. (B) For analyses, the cytotoxicity of all effector cell cultures was pooled for each target (N = 12 for each target). Shown is the mean ± SD of the cytotoxicity that was detected against target cells loaded with one of the peptides in a LDH release assay. One‐way ANOVA with Bonferroni's Multiple Comparison Test: *P < 0.05, **P < 0.01

On day 14 of stimulation, in vitro response of stimulated cells was analyzed in a cytotoxicity assay by measuring LDH release of peptide loaded T2/mHLA‐A*24:02 cells. Therefore, stimulated effector cells were incubated with target cells loaded with all different peptides (Figure 6A). The AQ14 loaded target cells showed significant higher LDH release compared to target cells without peptide and to all other peptides, independently of the peptide that was used for stimulation (Figure 6B). AF15 or VL9 loaded target cells were not lysed more often than target cells without peptides. Only for effector cells stimulated with VL9, the cytotoxicity against target cells reached the same level for VL9 as for AQ14.

### 3.3 The AQ14 peptide can only be found with a low ranking score in peptide prediction databanks

Because most studies that involve anti‐viral T‐cell stimulation protocols utilize predicted HLA restricted peptides for their experiments, we investigated whether the HCMV peptides that we found to be naturally selected and presented by HLA‐A*24:02 in BJ cells would also be predicted by those bioinformatic tools. The naturally isolated HCMV peptides were between 14 and 31 aa in length, but for HLA class I alleles the predicted peptides in the databases are limited to a maximum of 14 aa. Therefore, we searched in the prediction tools only for the isolated 14‐meric peptides. The source protein sequence of AQ14, transcription factor IE2, was entered in the different databases and the output for HLA‐A*24:02 binding was analyzed for the sequence of AQ14. In the IEDB database, the results are listed according to the percentile rank of the peptide with small numbers for high affinity. In this database, AQ14 was ranked with a percentile rank of 97.0 and an ANN IC50 value of 45 618.55 nM. In the NetMHCpan 4.0 database, the AQ14 peptide took the lowest rank for all 14‐meric peptides from IE2 with a score of 0.0000040 and a rank of 94.6038%.

### 3.4 Molecular modeling of the structure of HLA‐A*24:02 with predicted and isolated peptides

**Figure 7:**
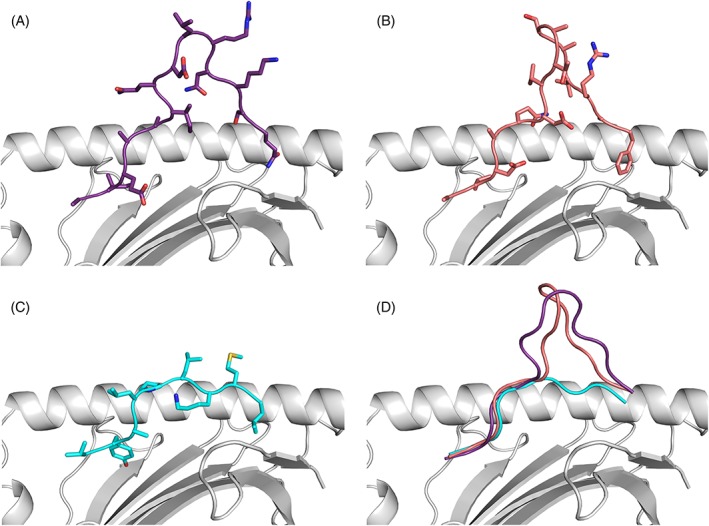
Model of HLA‐A*24:02 with human cytomegalovirus (HCMV)‐derived peptides. Modeling was performed using the structure of A*24:02 (PDB 3WL9) in Rosetta FlexPepDock. A: AQ14 peptide. B: AF15 peptide. C: VL9 peptide. D: Overlay of AQ14, AF15 and VL9 peptide

We performed computational modeling of three peptides (Figure 7): VL9, a predicted and commonly used T‐cell therapeutic peptide and the AF15 and AQ14 peptides identified in this work. Modeling was performed using the structure of A*24:02 (PDB 3WL9) in Rosetta FlexPepDock. Overall the structure of the HLA heavy chain in all three models shows no significant structural alterations,, with one exception of subtle differences in rotamer conformations for residues Q155, Q156, and Y159 in the AQ14 structure. Additional rotamer conformation changes were also observed in several peripheral residues not directly contacting the peptide, E62, K66, R131, E154, and R157. The structures of the three modeled peptides are characteristically anchored at their termini and show as would be expected completely different conformations. While VL9 lies flat in the peptide binding groove in the characteristic manner of canonical 9 to 11‐meric peptides the central portions of the AF15 and AQ14 bulge out of the peptide binding groove with the N‐ and C‐terminal residues anchoring the peptides in the cleft. In the long peptides, the loop of AF15 shows a distinct β‐hairpin secondary structure while the AQ14 peptide adopts a wider more open conformation.

## 4 DISCUSSION

The prediction of peptide presentation requires a very comprehensive knowledge for the function of different HLA variants and should consider alterations in the proteome and ligandome under different pathological conditions. The imperative for this kind of comprehensive information becomes obvious when T‐cell therapies have to be established to overcome infections under immunocompromised settings.

The reactivation of HCMV in immunocompromised patients is a feared complication. Different methods for treatment with donor T‐cells specific for viral peptides are now being developed; however, these applications are usually based on predicted peptide sequences. The present study was designed to improve the understanding of peptide selection and the selection of candidate peptide sequences. These findings represent a proof of concept that can be used in developing candidate peptides from other HLA class I alleles and show the limitations in bioinformatics approaches to peptide selection.

The *H. sapiens*‐derived peptides from infected and uninfected cells were compared to analyze differences in the peptidome. Only a small number of peptides were shared between uninfected‐ and HCMV‐infected cells. This implies that peptide presentation is not only impaired because HLA cannot be transported to the cell surface, but that all steps necessary to produce a complete, stable HLA molecule can be disrupted or at least altered during pathogenic episodes. However, one has to bear in mind that in this study the peptide repertoire of a tapasin‐independent allele was examined. In case of tapasin‐dependent alleles the results might be different.

This work shows to our knowledge for the first time naturally HCMV‐derived HLA‐A*24:02 restricted peptides isolated from HLA class I molecules in HCMV infected cells. The peptides are derived from HCMV proteins that are specific for different time points in the infection profile. The transcription and translation of HCMV proteins in the lytic cycle is a precisely regulated cascade of processes^65^, ^66^ and is divided into three different phases: Phase I starts immediately after infection with expression of transcription factors and immune escape proteins (IE genes). In the next phase, a number of different proteins are expressed, including proteins for viral replication (early [E] genes). In the last phase, expression of proteins required for formation of viral particles are expressed (late [L] phase). Infected cells in the body will be in different viral replication phases at the point of therapy; that should also be reflected in the selection of peptides for T‐cell therapies. The naturally processed, selected and presented peptides isolated in the present study contained peptides of different time points with most peptides from E‐L because many peptides were derived from UL22A. To date, peptides from pp65, a late phase protein, are commonly used for T‐cell therapies.^67^ To catch infected cells in all viral replication phases, a combination of peptides from different time points would be an ideal procedure; the described peptide AQ14, derived from an IE protein, represents a perfect additional peptide that could be combined with existing therapeutic peptides.

The HCMV‐derived HLA‐A*24:02 peptides described here are of extraordinary length and show altered anchor positions compared to those described in previous work. It becomes obvious that HCMV infected cells present peptides with a different binding motif than uninfected cells because of the many interventions of immune evasion proteins with the peptide loading complex. It might also be reasonably assumed that the peptide selection process in HCMV infected and uninfected cells is not comparable. In addition, published peptide anchors are derived from B‐lymphocytes and might differ in different cell types. Several studies have proposed that the concept of selective and specific anchor positions should be rethought in terms of permitting more amino acids at anchors, because adjacent amino acids might compensate suboptimal binding and because rare amino acids are underestimated.^68^, ^69^ Especially in HCMV infected cells peptide loading is disrupted by viral immune evasion mechanisms and the characteristics of presented peptides can change as we demonstrate in this study. Desmet et al.^68^ also proved that HLA‐A*24:02 is able to bind peptides not corresponding to the published anchor motifs.

Although canonical HLA peptide sequences are usually 8‐11 aa long we^70^ and others^49^, ^50^, ^71^, ^72^ have shown that many larger peptides are able to be selectively presented by HLA molecules. To date, none of the current bioinformatic tools that predict CTL epitopes take these long peptides into account and structural data are limited to a handful of structures with 12 to 16‐meric peptides.^73^ Modeling of HLA‐peptide complexes using Rosetta Flexpepdock has proven to be an accurate method for structural visualization. The bulged structures of extremely long peptides often illustrate conformational restraints and this has been well studied through extensive bioinformatic analysis.^74^ In the modeled peptide structures the AF15 peptide illustrated a rigid β‐hairpin structure that is reminiscent of the recently published HLA‐A*02:01 FNLKD 15‐meric peptide structure.^73^

In mice, a recent study provided insight in another factor that could play a role in immunogenicity of peptides.^75^ Testing different MCMV peptides, peptides from C‐terminal ends of proteins resulted in stronger T‐cell responses after vaccination. This effect might also hold true for HCMV peptides presented by HLA class I molecules after infection, since the C‐terminal AQ14 peptide elicited higher cytotoxicity than the AF15 peptide that is located near the N‐terminus. This might also be the reason why all effector cells, independently from the peptide that was used for stimulation, achieved the highest cytotoxicity against HLA‐A*24:02 target cells bound to AQ14, although, the HLA class I genotype of the cells differ. Only VL9 stimulated effector cells showed the same level of cytotoxicity against target cells loaded with VL9. The VL9 peptide is in contrast to AQ14 in peptide prediction tools among the high rated peptides. However, the cytotoxicity test shows that the highest rated peptides are not necessarily the most immunogenic peptides. Furthermore, the analysis of marker expression of stimulated CD8^+^ T‐cells showed no correlation with cytotoxicity.

Based on these results, it becomes obvious that algorithms of peptide prediction tools are not sufficient to predict the most immunogenic peptides. Changes in selection of peptide length and to restrictions of peptide anchors have to be made if those tools should be used to select peptides for T‐cell therapies. The HLA class I binding groove is obviously much less restricted in peptide binding than proposed before, especially in case of impaired peptide presentation due to infection. Taking all these results into account might improve T‐cell therapies with non‐responders at the present stage leading the way.

## CONFLICT OF INTEREST

The authors have declared no conflicting interests.

## AVAILABILITY OF DATA

The data that support the findings of this study are available on request from the corresponding author. The data are not publicly available due to privacy or ethical restrictions.

## Supporting information


**FIGURE S1** Monitoring of sHLA‐A*24:02 expression in the supernatant of uninfected and HCMV infected *BJ* cells. Supernatant of sHLA‐A*24:02 transduced *BJ* cells was analyzed weekly by sandwich‐ELISA. Exemplary results from uninfected cells are given in black, results from HCMV infected cells are given in gray.

**FIGURE S2** Comparison of uninfected and infected BJ cells after 7 days post‐infection. The CPE was checked after 7 days. Infected cells detached from the surface, showed a swollen cytopathology and partly died.

**FIGURE S3** pp65 expression in *BJ*/sHLA‐A*24:02 cells infected with HCMV over time. To validate infection of cultivated BJ cells, the expression of the HCMV phosphoprotein 65 and the cell viability (7‐AAD) was measured over 7 days.
