# Supplementary material for: Releasing the concept of HLA‐allele specific peptide anchors in viral infections: A non‐canonical naturally presented human cytomegalovirus‐derived HLA‐A*24:02 restricted peptide drives exquisite immunogenicity
Source: HLA. 2019 Apr 14;94(1):25–38. doi: 10.1111/tan.13537 (PMC6593758; doi:10.1111/tan.13537)
Supplement: Supplementary file 1 — FIGURE S1 Monitoring of sHLA‐A*24:02 expression in the supernatant of uninfected and HCMV infected BJ cells. Supernatant of sHLA‐A*24:02 transduced BJ cells was analyzed weekly by sandwich‐ELISA. Exemplary results from uninfected cells are given in black, results from HCMV infected cells are given in gray. [file TAN-94-25-s001.docx]

**
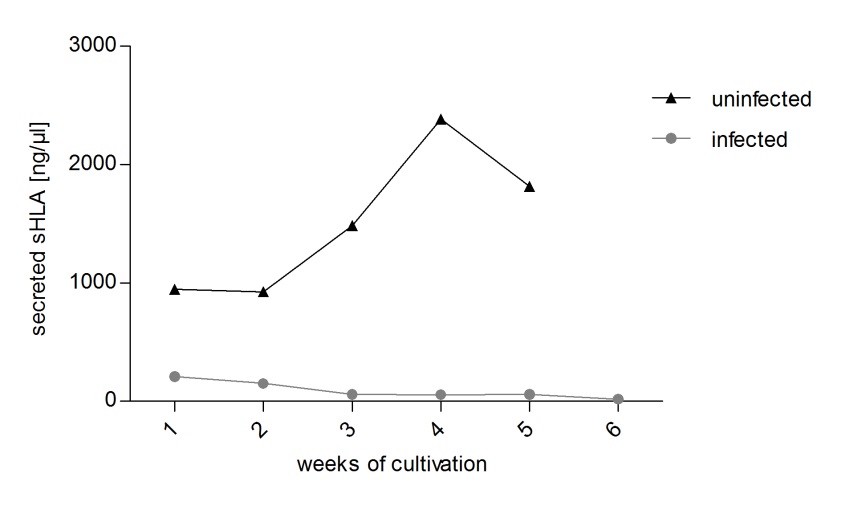
**

**Supplemental Figure 1: Monitoring of sHLA-A*24:02 expression in the supernatant of uninfected and HCMV infected *BJ* cells.** Supernatant of sHLA-A*24:02 transduced *BJ* cells was analyzed weekly by sandwich-ELISA. Exemplary results from uninfected cells are given in black, results from HCMV infected cells are given in grey.
