# Supplementary material for: Releasing the concept of HLA‐allele specific peptide anchors in viral infections: A non‐canonical naturally presented human cytomegalovirus‐derived HLA‐A*24:02 restricted peptide drives exquisite immunogenicity
Source: HLA. 2019 Apr 14;94(1):25–38. doi: 10.1111/tan.13537 (PMC6593758; doi:10.1111/tan.13537)
Supplement: Supplementary file 2 — FIGURE S2 Comparison of uninfected and infected BJ cells after 7 days post‐infection. The CPE was checked after 7 days. Infected cells detached from the surface, showed a swollen cytopathology and partly died. [file TAN-94-25-s002.docx]

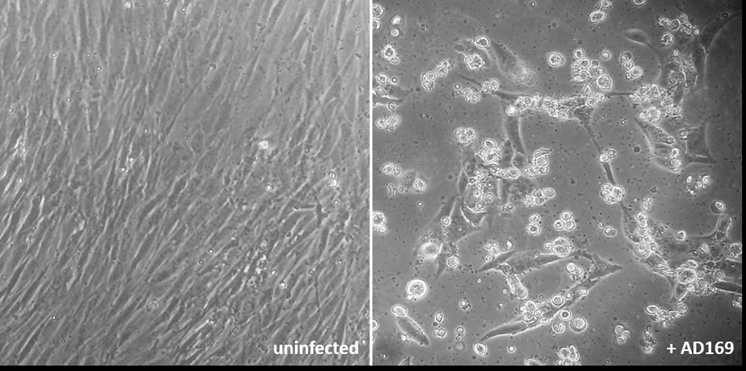


**Supplemental Figure 2: Comparison of uninfected and infected BJ cells after 7 days post-infection.** The CPE was checked after 7 days. Infected cells detached from the surface, showed a swollen cytopathology and partly died.
