# Supplementary material for: Releasing the concept of HLA‐allele specific peptide anchors in viral infections: A non‐canonical naturally presented human cytomegalovirus‐derived HLA‐A*24:02 restricted peptide drives exquisite immunogenicity
Source: HLA. 2019 Apr 14;94(1):25–38. doi: 10.1111/tan.13537 (PMC6593758; doi:10.1111/tan.13537)
Supplement: Supplementary file 3 — FIGURE S3 pp65 expression in BJ/sHLA‐A*24:02 cells infected with HCMV over time. To validate infection of cultivated BJ cells, the expression of the HCMV phosphoprotein 65 and the cell viability (7‐AAD) was measured over 7 days. [file TAN-94-25-s003.docx]

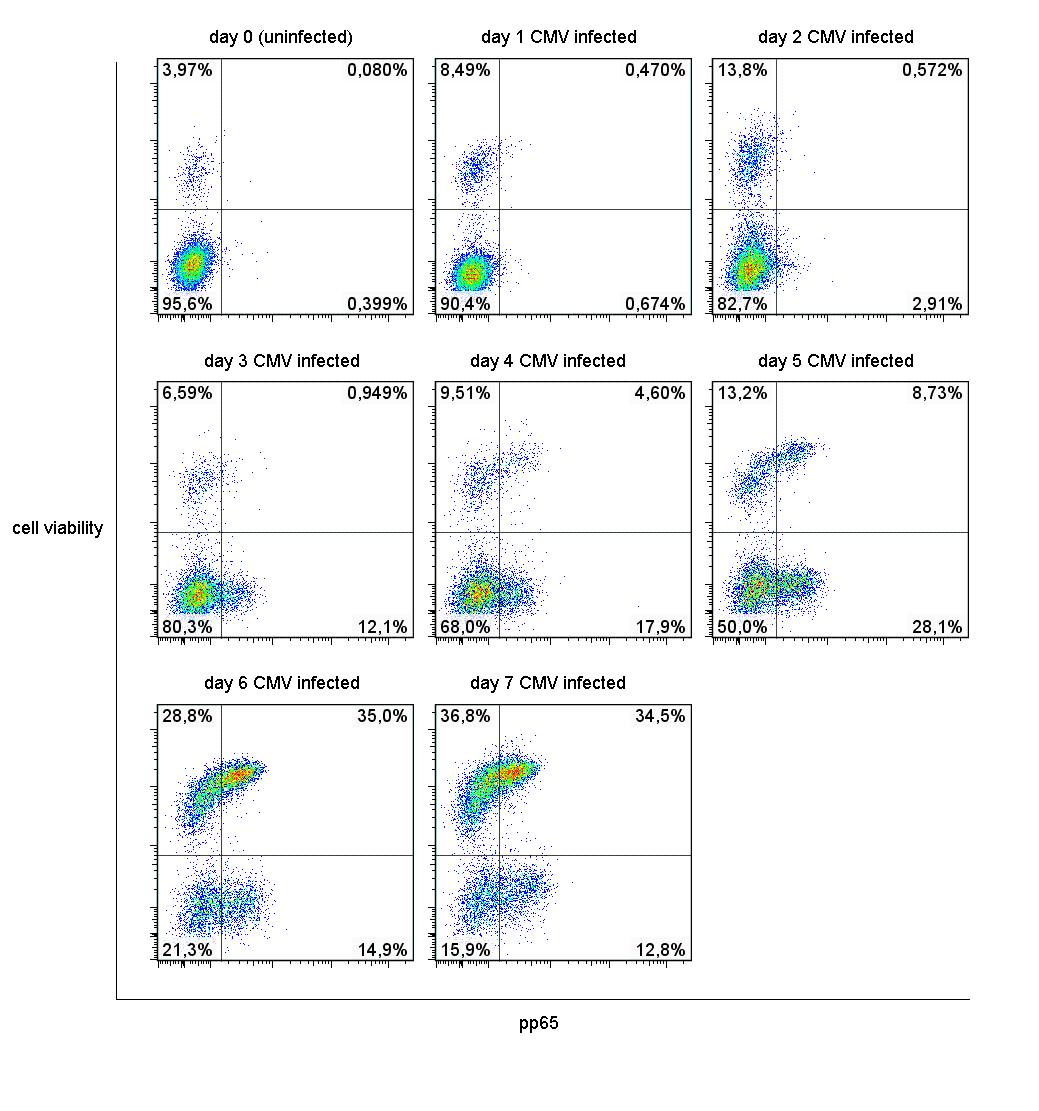


**Supplemental Figure 3: pp65 expression in BJ/sHLA-A*24:02 cells infected with HCMV over time.** To validate infection of cultivated BJ cells, the expression of the HCMV phosphoprotein 65 and the cell viability (7-AAD) was measured over 7 days.
